# Frogs, Glorious Frogs (1999)

**DOI:** 10.3201/eid0506.AC0506

**Published:** 1999

— Amanda Hyatt

This issue was originally published without an accompanying cover story. 

**Figure:**
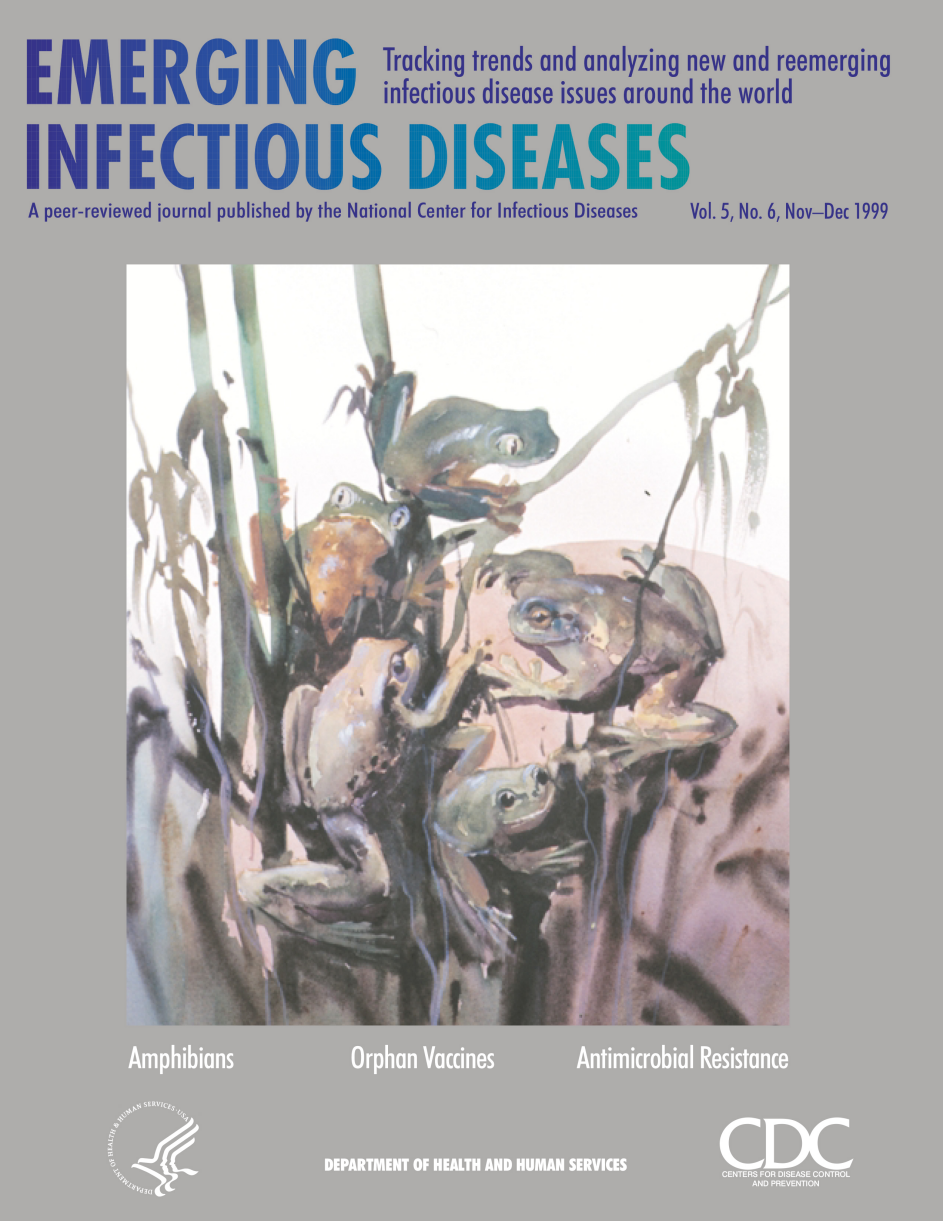
**Amanda Hyatt. Frogs, Glorious Frogs (1999).** Reprinted with permission of the artist, Geelong, Australia.

